# First Molecular Survey on *Anaplasma phagocytophilum* Revealed High Prevalence in Rural Dogs from Khuzestan Province, Iran

**Published:** 2019

**Authors:** Hossein HAMIDINEJAT, Somayeh BAHRAMI, Bahman MOSALANEJAD, Sharareh PAHLAVAN

**Affiliations:** 1. Department of Parasitology, Faculty of Veterinary Medicine, Shahid Chamran University of Ahvaz, Ahvaz, Iran; 2. Department of Clinical Sciences, Faculty of Veterinary Medicine, Shahid Chamran University of Ahvaz, Ahvaz, Iran

**Keywords:** *Anaplasma phagocytophilum*, Dog, Iran, Nested-PCR

## Abstract

**Background:**

Anaplasmosis due to *Anaplasma phagocytophilum* is an important tick-borne zoonotic disease, which affects dogs, horses, cattle and human as well. This study aimed to probe the existence of this organism by means of molecular biology techniques for the first time in rural dogs of Khuzestan province, Southwestern Iran.

**Methods:**

During Sep 2014 to Apr 2015 blood samples of 103 apparently healthy rural dogs (60 males) were collected for *A. phagocytophilum* detection by light microscopical examination of Giemsa stained slides and Nested PCR on a fragment of 16S rRNA gene.

**Results:**

From the examined slides, 11.65% were positive for *A. morulae* while 57.28% of infection was revealed by Nested PCR method. There was no statistical difference between ages and sexes of dogs and infection in molecular survey of *A. phagocytophilum*.

**Conclusion:**

Molecular prevalence of *A. phagocytophilum* was noticeably high. It may cause the incidence of disease in human population.

## Introduction

*Anaplasma phagocytophilum* (Order, Rickettsiales; Family, Anaplasmataceae) is an emerging zoonotic tick-borne rickettsia, with veterinary and public health importance worldwide (1, 2). The organism is predominantly transmitted by *Ixodes* spp*.* hard ticks, but other questing hard ticks might act as vectors (3–5). *A. phagocytophilum* is closely related to the Gram-negative bacteria *Ehrlichia* and can be transmitted to wide range of animals causing granulocytic anaplasmosis that especially produce clinical signs in dogs, horses, cattle and human as well (6,7). Unlike *A. marginale* and *A. centrale*, which settle in erythrocytes, *A. phagocytophilum* prefers leukocytes as its favorite habitat named in this position as morulae (8). *A. phagocytophilum* causes human granulocytic anaplasmosis (HGA) that ranges in severity from asymptomatic seroconversion to a mild or severe febrile illness. In rare instances, severe disease can result in organ failure or death.

Nonspecific signs and symptoms including acute onset of fever, headache, malaise and myalgia are typical, even in severe infection. Less common symptoms include nausea, abdominal pain, diarrhea, and cough (3). HGA is considered a zoonosis of increasing concern (9–11). The disease manifests sub-clinical and occasionally fatal features and responsible for important economic losses to livestock industry (12,13). Afflicted dogs may develop either clinically acute febrile illness distinguished by fever, polyarthritis, thrombocytopenia, leucopenia, depression and anorexia or represent no distinct noticeable abnormalities and specific clinical signs (6, 12). However, some clinical features including lameness, coughing, polydipsia, intermittent vomiting, and hemorrhages are considered as less frequent sequela of this tick-borne fever in dogs (8).

In large-scale surveys, determination of *A. phagocytophilum* is based upon three distinct procedures, generally included microscopy by preparing thin layer Romanowsky-stained peripheral blood, serology and molecular techniques (8, 9, 12). Therefore, we utilized microscopy to detect morulae within neutrophils and specific Nested PCR to gain results that are more accurate.

Regardless of little scattered studies about *A. phagocytophilum*, there is no molecular-based documented data concerning the occurrence of this bacterium in dogs of Iran. Therefore, this study aimed to evaluate the prevalence of *A. phagocytophilum* infection in apparently healthy rural dogs from Khuzestan Province, Southwestern Iran by molecular and staining methods and to compare results from mentioned methods.

## Materials and Methods

### Sampling

During Sep 2014 to Apr 2015, 103 rural apparently healthy dogs (which consisted of 76 dogs equal and less than three years old and 27 dogs more than three years old and 60 male and 43 female) from Khuzestan Province in south-west of Iran were examined for *A. phagocytophilum* infection. Samples originated from four geographical regions within Khuzestan Province from villages around Shush (in North), Abadan (in South), Ramhormoz (East) and Hamidieh (South). Dogs were restrained and from each animal 2 ml of peripheral whole blood of saphenous or cephalic vein were taken and transferred to tubes containing coagulant for molecular investigation and Romanowsky-staining. In some cases, intra-muscular acepromazine (0.15 mg/kg) and ketamine (15 mg/kg) injection were used for relaxing of dogs.

The animals were handled according to the recommendation of the Ethics Committee, Shahid Chamran University of Ahvaz, Ahvaz, Iran (Ethical Approval Number 2/3/93, 23 July 2014).

### Microscopy

From each sample, thin blood smear was prepared following by methanol fixation and to stain with 10% of Giemsa (Merck, USA) for 30 min. Slides were then examined for detection of morulae of *A. phagocytophilum* using ×1000 magnification of light microscopy by searching all area of slide.

### PCR

DNA was exploited by application of the genomic DNA extraction Kit (Cinnagen, Iran). Species specification was accomplished by Nested PCR according to amplification of 16S rRNA gene conserved for all *Anaplasma* species. PCR protocol and primers were adopted from description (14). Briefly, amplification was performed in 25 μl reaction volumes including 5 μl of DNA template, 5 pmol of forward and reverse primers (each 1 μl), 12.5 μl of master mix (Ampliqon, Denmark) containing 3 mM MgCl_2_, 0.4 mM of each dATP, dCTP, dGTP and dTTP and 0.08 U/ml Taq DNA polymerase in reaction buffer. Forward and reverse genus-specific primer sequences were 5′-AGAGTTTGATCCTGGCTCAG-3′ and 5′-AGCACTCATCGTTTACAGCG-3′, respectively. Positive samples showed a band of approximately 781 bp. Nest 2 reactions were carried out on products of prime PCR by forward and reverse primers of 5′-GCAAGCTTAACACATGCAAGTC-3′ and 5-GTTAAGCCCTGGTATTTCAC-3′ for Nested PCR to confirm the *Anaplasma* spp. and 5′-CTTTATAGCTTGCTATAAAGAA-3′ and 5′-GTTAAGCCCTGGTATTTCAC-3′ for specific Nested PCR to confirm *A. phagocytophilum*. Positive samples showed a band of approximately 543 and 504 bp in Nested PCR and specific Nested PCR, respectively. The thermal program of all PCRs was as follows: 95 °C for 5 min, 35 cycles of 94 °C for 45 sec, annealing at 56 °C for 45 sec, and 72 °C for 45 sec, followed by a final extension step at 72 °C for 5 min.

### Statistical analyses

The data were correlated with sex and age of the dogs and the statistical significances of their relation or independence were evaluated by Chi-square test (χ^2^), Fisher’s exact test and logistic regression with 95% of confidence level. Analyses were performed by SPSS 18.0 software (Chicago, IL, USA) for Windows 8, with a *P*-value <0.05 as statistically significant. Confidence limits for the proportions were established by exact binomial test with 95%.

## Results

**Fig. 1:**
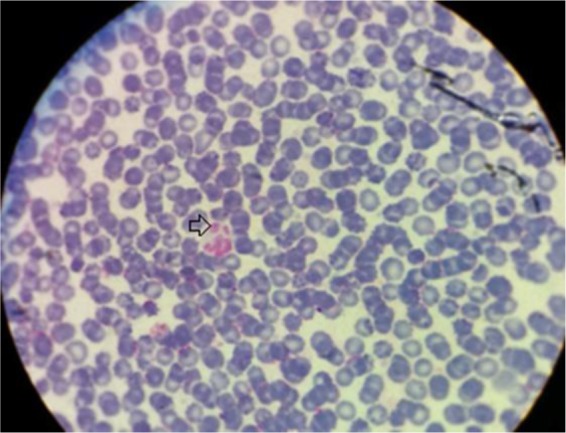
Morulae of *Anaplasma phagocytophilum* in a neutrophil of a dog (Arrow)

From 103 prepared slides of examined rural dogs, 11.65% (12 from 103) were microscopically positive for morulae of *Anaplasma* (Fig. 1).

**Fig. 2:**
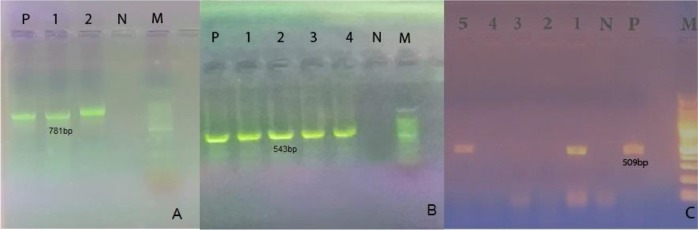
Agarose gel electrophoresis of PCR of 16S rRNA gene (A), Nested PCR (B) and Specific Nested PCR (C) products. A. 1,2: positive samples for *Anaplasma* spp. P: positive control, N: negative control (781 bp). B. 1–4: positive samples for *Anaplasma* spp. P: positive control, N: negative control (543 bp). C. 1,5: positive samples for *A. phagocytophilum*. P: positive control, N: negative control (509 bp). M is a 100 bp ladder

All of the approximately 781bp bands amplified in prime PCR were confirmed by detecting of expected 544 bp length bands (Fig. 2). Specific Nested PCR revealed high prevalence of infection, that specific band with 509 bp length bands were exhibited in 57.28% of cases (59 of 103) in electrophoresis (Fig. 2). Molecular method diagnosed more infected dogs than staining method.

In terms of sex, there was no significant difference between the prevalence percentage of infection in males and females (*P*=0.58). Age was not identified as a risk factor for *A. phagocytophilum* infection in this study (*P*=0.5). There was not a significant geographical variation in the prevalence of *A. phagocytophilum* infection, ranging from 45.83% in Shush to 69.23% in Ramhormoz (CI, 2.46%–76.82%). The odds of infection in dogs in west were 2.6 times greater than in animals kept in north (Table 1).

**Table 1:** Prevalence of *A. phagocytophilum* in dogs from Khuzestan province, southwest of Iran and related factors

***Factor***	***No. examined***	***Positive***	***%***	***P-value***	***OR***	***CI 95%***
Prevalence	103	59	57.28	-	-	-
Gender						
Male	60	33	55	0.58	1.25	0.56–2.7
Female	43	26	60.46			
Age						
≤3 yr old	76	45	59.2	0.5	1.31	0.54–3.19
>3 yr old	27	14	51.8			
Locality						
Shush	24	11	45.83	-	-	-
Abadan	26	15	57.69	0.53	1.6	0.52–5.15
Ramhormoz	26	18	69.23	0.42	1.8	0.58–5.5
Hamidieh	27	15	55.5	0.55	2.6	0.83–8.45

## Discussion

Clinical manifests of *A. phagocytophilum* are not always pathognomic, therefore diagnosis of this infection is basically based upon paraclinical aspects of the infection. For this purpose, many diagnostic approaches including microscopy to recognize morulae in leukocytes, different serologic procedures and tracing DNA of rickettsia from blood, buffy coat, bone marrow or spleen are developed (8). Intra-leukocytic morulae are indiscernible from those of *Ehrlichia* species. On the other sides, serology is along with cross-reacting phenomenon, seronegative response in the primary phase and indiscrimination between recent and former infections, since anaplasmosis due to *A. phagocytophilum* is normally a self-limiting infection (15, 16). Therefore, in such cases sensitive and specific molecular recognition of the organism is method of choice (15). Among the different targeted genes for amplification in PCR, 16S rRNA gene with highly variable nucleotide region appears more reliable as a matter of *A. phagocytophilum* detection (4, 17,18). However, we applied both specific PCR and microscopy to establish a comparison on this issue.

In Iran, for the first-time detection of *A. phagocytophilum* was reported (14). 16S rRNA and MSP4 genes of *A. phagocytophilum* were analyzed by Nested-PCR method and overall 1.08% (4/370) of domesticated small ruminants was positive for *A. phagocytophilum* infection (19).

By molecular approach, we determined the infection rate of 57% in dogs, whereas microscopy indicated 11.65% of infection. In 18% of dogs from Tehran *A. phagocytophilum* morula as forms were detected (20). Four percent of *A. phagocytophilum* infection was found in dogs from Turkey by molecular method (21). Surprisingly, according to current work molecular prevalence of *A. phagocytophilum* in the zone of study is considerably high in comparison with other countries and this infection is hyper enzootic in this area. In a Brazilian survey, 6.03% of dogs were infected with this rickettsia (22). In central Italy, only 0.9% of prevalence were in hunter dogs (23). Latter two studies were performed by PCR based on 16S rRNA gene.

There was a wide gap between results of two applied procedures in the present survey (11.65% vs 57.28%), in which microcopy was very less sensitive than PCR. Such as all the infective organisms of circulating cells especially in low bacteremia period, detecting of *A. phagocytophilum* in neutrophils requires a professional microscopist thereupon maybe we missed these rickettsia in our slide probing with considering the short time of bacteremia pick of *A. phagocytophilum* (7).

All the examined dogs in this investigation were apparently healthy. Although based on living circumstances in rural districts in southwest of Iran, dogs are not always in very tight relation with owners so one recent illness may miss from attention. These animals have almost elapsed their whole life in such a condition in villages and unlike pets or even stray dogs are in close association with other domestic animals, which are hosts of numerous hard ticks and *A. phagocytophilum* in pastures (19).

In accordance with high prevalence of *A. phagocytophilum* infection in current survey, this infection is more likely present in its sub-clinical or silent mode in study. Unanimous with our discussion, most of *A. phagocytophilum* infections are sub-clinical in dogs of Germany (24). In dogs, pathogenicity of *A. phagocytophilum* is *per se* related to different factors such as concomitant infections, animal housekeeping, nutrition, immunity and variant of rickettsia. In Iran, rural dogs are in close confrontation with villagers. These domesticated animals are hardly ever considered for spraying or utilizing of pesticide compounds by owners and tick infestation is a current problem in them. On the other hand, unfortunately, there is no confirmative data about *A. phagocytophilum* infection of human population from Khuzestan, the area of this study, but considering that human granulocytic anaplasmosis is globally discussed as an emerging important zoonosis, and high-contaminated population of dogs is most probably increasing the incidence of disease in at risk public. However, different variants of *A. phagocytophilum* had inclination to distinct hosts. Correlation among host tropism and pathogenicity is also another issue which may impress the zoonotic potential of this organism (8, 25). As a zoonosis, the variants of *A. phagocytophilum* among domestic and wild life cycle of the organism should be crucially considered.

## Conclusion

Molecular prevalence of *A. phagocytophilum* was noticeably high in dogs form Iran. It may cause the incidence of disease in human population.
